# Disruption of biological membranes by hydrophobic molecules: a way to inhibit bacterial growth

**DOI:** 10.3389/fmicb.2024.1478519

**Published:** 2025-01-08

**Authors:** Alejandra Gabriela Valdez-Lara, Ángela M. Jaramillo-Granada, Daniel Ortega-Zambrano, Eristeo García-Marquez, Jorge Alberto García-Fajardo, H. Mercado-Uribe, J. C. Ruiz-Suárez

**Affiliations:** ^1^Centro de Investigación y de Estudios Avanzados del Instituto Politécnico Nacional Unidad Monterrey, Apodaca, Nuevo León, Mexico; ^2^Centro de Investigación y Asistencia en Tecnología y Diseño del Estado de Jalisco Subsede Noreste, Apodaca, Nuevo León, Mexico

**Keywords:** *E. coli*, *S. aureus*, liposomes, propofol, CBD

## Abstract

With antibiotic resistance increasing in the global population every year, efforts to discover new strategies against microbial diseases are urgently needed. One of the new therapeutic targets is the bacterial cell membrane since, in the event of a drastic alteration, it can cause cell death. We propose the utilization of hydrophobic molecules, namely, propofol (PFL) and cannabidiol (CBD), dissolved in nanodroplets of oil, to effectively strike the membrane of two well-known pathogens: *Escherichia coli* and *Staphylococcus aureus*. First, we carried out calorimetric measurements to evaluate the effects of these drugs on model membranes formed by lipids from these bacteria. We found that the drugs modify their transition temperature, enthalpy of cohesion, and cooperativity, which indicates a strong alteration of the membranes. Then, inhibition of colony-forming units is studied in incubation experiments. Finally, we demonstrate, using atomic force and fluorescence microscopy, that the drugs, especially propofol, produce a visible disruption in real bacterial membranes, explaining the observed inhibition. These findings may have useful implications in the global effort to discover new ways to effectively combat the growing threat of drug-resistant pathogens, especially in skin infections.

## 1 Introduction

Antimicrobial resistance (AMR) increases every day, a phenomenon that is a global health problem mainly in immunocompromised cancer patients (Bodro et al., 2014; Gudiol and Carratalà, 2014), organ transplant surgeries (Ye et al., 2014), infections in chronic wounds (Kawano et al., 2020), and among others (Dadgostar, 2019). It is expected that in the serious scenario where antibiotics become ineffective, millions of deaths could produce a pre-antibiotic era (Davies and Davies, 2010). Investigations reveal that in 2019, 1.27 million deaths were caused by antimicrobial resistance, and 4.95 million were indirectly associated with it (Murray et al., 2022). In addition to these human losses, the Center for Disease Control and Prevention estimated that in the United States, the economic impact is 55 billion dollars per year, considering not only public health spending but also the loss of productivity (Dadgostar, 2019). Indeed, AMR is not only an exclusive public health problem but may trigger economic consequences worldwide. If precautions are not taken, human deaths are expected to reach 10 million by 2050, resulting in a global cost of 100 billion dollars (O'Neill, 2016).

The World Health Organization (WHO) has published a list of 12 pathogens in urgent need of new antibiotics (www.who.int/news/item/27-02-2017-who-publishes-list-of-bacteria-for-which-new-antibiotics-are-urgently-needed). Unfortunately, studies on new antibiotics are scarce, as in 2020 there were only 135 preclinical projects in development worldwide (Theuretzbacher et al., 2020).

It is known that the plasmatic membrane is crucial for the correct functioning of a cell (Derby and Gleeson, 2007; De Rosa et al., 2007; Adibhatla and Hatcher, 2008). The membrane protects it, maintains the balance between the inside and outside, regulates cell traffic, and participates in cellular responses (Malanca and Camici, 1989; Marza et al., 2002; Ammendolia et al., 2021). In eukaryotic cells, alterations in membrane fluidity have been correlated with several diseases (Shinitzky, 1984; Sameni et al., 2018), while in procaryotic cells, fluidization has been found to cause disruption of the cell membrane (Royce et al., 2013). Thus, the membrane is a good target to hit because it could affect cell viability (Sudhahar et al., 2008), particularly when mutagenic bacterial populations are confronted with new scenarios to which they are not adapted (Salinas-Almaguer et al., 2022).

We propose propofol (PFL) and cannabidiol (CBD), two well-known molecules employed in many biophysics experiments to produce thermodynamic changes in lipid membranes (Momo et al., 2002; Tsuchiya, 2001; Perez et al., 2022), as a way to modify the elastic properties of cellular membranes. Propofol (2,6 diisopropylphenol), a small molecule (178.2 Da), is a general anesthetic with a high partition coefficient (logP 4.16). Since it has poor aqueous solubility, propofol is normally solubilized in oils and organic solvents. On the other hand, CBD is one of the 85 components of the cannabis plant without psychoactive effects and with multiple advantages such as anti-inflammatory, appetite stimulation in AIDS, and treatment for chronic pain, depression, anxiety, psychosis, and recently, antimicrobial effects (Blaskovich et al., 2021; Devinsky et al., 2017; McGuire et al., 2018). CBD is a much heavier molecule (314 Da) with a higher partition coefficient (logP 7.04).

In recent years, the FDA approved a new drug carrier emulsion of propofol based on soybean, but with many disadvantages such as an unstable emulsion in prolonged time and microbial contamination (Shevalkar et al., 2019). Antimicrobial activity of CBD against *N. gonorrhoeae* and *Moraxella catarrhalis*, both Gram-negative species, was recently reported (Blaskovich et al., 2021). The authors proposed that the effect of CBD is produced on the outer membrane of these bacteria, although they remark that the main effect is on the DNA, RNA, peptidoglycan, and lipid synthesis. In other words, they do not put any further consideration on the possible affectation of membrane integrity.

Due to its low water solubility and bioavailability in plasma or blood, the use of hydrophobic molecules is an interesting issue. Normally, as in the previous referred study (Blaskovich et al., 2021), the organosulfur compound dimethyl sulfoxide (DMSO) is used to dissolve both polar and non-polar molecules, although due to its toxic effect, the concentration cannot be higher than 0.5 % v/v. In recent years, nanoemulsions, used as drug carriers, have gained relevance because they can encapsulate molecules that are poorly soluble in water (Tayeb and Sainsbury, 2018; Li et al., 2012; Guzmán et al., 2021; Shakeel et al., 2012).

Nanoemulsions are colloidal dispersions composed of two immiscible liquids (i.e., oil-in-water or water-in-oil), with nanoscale-size droplets from 20 to 200 nm in diameter (Sagalowicz and Leser, 2010). Their small sizes have many advantages for drug delivery due to their high bioavailability and stability for an extended time. In addition, since the drug is immersed in nanodroplets it can be preserved for large periods (Karami et al., 2019; Sánchez-L0pez et al., 2019). A previous study concluded that a nanoemulsion of herbal plant oils modified the outer membrane of Gram-positive and Gram-negative strains (Krishnamoorthy et al., 2018). It seems that the plasma membrane, regardless of its composition, is the unspecific receptor for the molecules.

In this study, we investigate two pathogens that are included in the WHO list: *E. coli* and *S. aureus*. The first is a Gram-negative bacterium from the *Enterobactericeae* family, which shows resistance to carbapenems and also produces an extended-spectrum of beta-lactamase (ESBL). Its double lipid membrane, separated by a peptidoglycan layer, provides its protection to drugs. The second is a Gram-positive spherically shaped bacterium with a single membrane, whose natural habitat in humans is the skin and nasopharynx.

Before investigating the effects of PFL and CBD on *E. coli* and *S. aureus*, we performed calorimetric measurements of liposomes formed by lipids similar to those found in these bacteria. Having observed the strong effects that the aforementioned drugs produced on the membranes, we examined their effects on the growth of *E. coli* (K-12 MG1655) and *S. aureus* (Rosenbach Atcc 25923). A great correlation was found between the degree of alteration in the thermotropic profiles obtained by calorimetry and the effect on the growth of bacterial colonies. We also performed atomic force microscopy and fluorescence microscopy measurements to further evaluate the change in the morphology of the bacterial membranes.

## 2 Materials and methods

### 2.1 Reagents

1,2-dipalmitoyl-sn-glycero-3-phosphoethanolamine (DPPE) (99 %), 1,2-dipalmitoyl-sn-glycero-3-[Phospho-rac-1-glycerol] (DPPG) (99 %), 1',3'-bis[1,2-dimyristoyl-sn-glycero-3-phospho]-glycerol (CL), 2,6-Diisopropylphenol (PFL) (97%), olive oil (OO) (analytic grade), and chloroform (anhydrous, ≥99%, with 0.5 - 1.0% ethanol) were purchased from Sigma-Aldrich. N-octadecanoyl-D-erythro-sphingosylphosphoryl choline (sphingomyelin, brain porcine) (SM) was obtained from Avanti Polar Lipids. Texas Red dye (1,2-dihexadecanoyl-sn-glycero-3-phosphoethanolamine, triethylammonium salt) (TR-DHPE) was obtained from Invitrogen. Cannabidiol (CBD) (99%) (CAS: 13956-29-1) was purchased in CrescentCanna (New Orleans, USA). Very pure water (18.2 *MΩ*cm) was obtained from a Milli-Q IQ 7000 system.

The LB broth and agar (Miller) were made from casein peptone (Tryptone) (CAS: 91079-40-2), yeast extract (CAS: 8013-01-2), and sodium chloride (NaCl) (CAS: 7647-14-5), all from MCD Lab. The nutrient agar used was BD brand (SKU: 213000) and phosphate buffer (PBS, pH 7.4) (MDL: MFCD00131855).

### 2.2 Nanoemulsions preparation

CBD and PFL were separately dissolved in analytical grade olive oil (OO) to prepare stocks with a final concentration of 275 mg/mL. Thereafter, a mixture of the stocks and Milli-Q water was prepared to reach emulsions with concentrations of 1% v/v for both drugs and 3% v/v for CBD. A mixture of OO and water was prepared under the same conditions. To form the nanoemulsions, a vigorous manual agitation was first made. Then, we used a microfluidizer (Microfluidics M-110P, Massachusetts, USA) device at 100 and 150 MPa for three cycles per pressure. The temperature was controlled by adding ice to the heat exchanger container of the apparatus. Subsequently, they were stored in the fridge at 4 °C.

### 2.3 Preparation of bacteria-like membranes

The main lipid components of *E. coli* and *S. aureus* are the unsaturated lipids 1,2-dioleoyl-sn-glycero-3-phosphoethanolamine (DOPE), 1,2-dioleoyl-sn-glycero-3-phospho-(1-rac-glycerol) sodium salt (DOPG), and cardiolipin (CL). However, since DPPE and DPPG have the same polar heads as DOPE and DOPG, we used them to prepare the needed membranes, as previously proposed by Lombardi et al. (2017). The idea behind this exchange is to bring the transition temperatures into a window where they are more accessible to perform the measurements (from negative temperatures approximately –18 °C for the unsaturated lipids, to temperatures between 40 and 65 °C for the saturated ones). Two different liposomes were prepared, the first one with DPPE/DPPG (80/20 % mol), simulating the Gram-negative *E. coli* membrane (Lombardi et al., 2017), and the second with DPPG/CL (95/5 % mol), mimicking the Gram-positive *S. aureus* membrane (Malanovic and Lohner, 2016).

The liposomes were prepared at a final concentration of 5 mM. Lipids were dissolved in 1.2 mL of chloroform inside a clear amber scintillation vial. The solvent was evaporated first with a nitrogen flux and then using a degassing station (TA instruments, New Castle) at 55 °C for 1.5 h with agitation at 130 rpm to remove any traces of chloroform. The film was subsequently re-hydrated with Milli-Q water at 60 °C with agitation (230 r.p.m.) for 2 h; the final formed lipid vesicles were multilamellar vesicles (MLVs).

### 2.4 Sample preparation for antimicrobial activity

For this study, *E. coli* (K12-MG1655) and *S. aureus* (Rosenbach Atcc 25923) strains were selected and pre-inoculums cultivated in Luria-Bertani (LB) medium at 37 °C until an optical density (OD) of 0.4 is reached, which corresponds to the exponential growth phase. This occurs approximately in 18 h. Thereafter, a series of four dilutions is made. The first dilution was prepared with a relation of 1:100 (0.2 mL bacteria and 20 mL of LB medium). Then, three consecutive dilutions are made with a ratio of 1:1, also incubated and mixed until the same absorbance (0.4 OD) is obtained. The last dilution was centrifuged at 4,000 r.p.m. for 10 min; then, the supernatant is eliminated, and the pellet was recuperated and re-suspended in a 10 mM phosphate buffer saline (PBS). This is repeated three consecutive times.

### 2.5 Differential scanning calorimetry

A total of 500 μL of large unilamellar vesicles (LUVs) and 500 μL of the nanoemulsion were mixed for a total of 3.7 mg/mL lipid concentration and 2.75 mg/mL (1% v/v) or 8.25 mg/mL (3% v/v) of the drug (PFL or CBD). Then, all of the samples stayed at room temperature for 15 min and degassed at –635 mmHg before calorimetric analysis in a differential scanning calorimeter (Nano DSC, TA Instruments, New Castle). Each sample was scanned in heating mode. After 600 s of equilibrium time, the profile was recorded at a rate of 1 °C/min and cell pressure of 3 bar. Three independent experiments were performed.

### 2.6 Characterization of nanoemulsions

Dynamic light scattering (DLS) was used to determine the size distribution of the nanoemulsions. They were diluted by 10x before carrying out size and zeta potential measurements in a zeta sizer (Nano ZSP, Malvern Instruments, United Kingdom). The laser wavelength and detector angle location were 633 nm and 173°, respectively. The instrument recorded intensity fluctuations, which were then analyzed using the Stokes-Einstein equation *R* = *K*_*B*_*T*/6π*ηD*, with R, *K*_*B*_, T, η, and D being the hydrodynamic radius, Boltzmann constant, temperature, dynamic viscosity, and diffusion coefficient, respectively. Zeta potential was calculated from the electrophoretic mobility employing Smoluchowski equation μ = ϵζ/η, where ϵ is the dielectric constant and η the dynamic viscosity. All measurements were conducted at 25°C, and each measurement was repeated at least three times °C.

### 2.7 Colony-forming unit assay

To find the amount of dead cells, four dilution series are performed 1:10 (100 μL suspended bacteria and 900 μL nanoemulsion) for each case (OO 1% v/v, OO 3% v/v, CBD-OO 1 and 3% v/v, and PFL-OO 1 % v/v). Next, an aliquot of 100 μL was placed in the petri dish, to spread it evenly across the surface of the LB agar. The cultures were incubated for 12 h at 37°C. The countable ranges were between 20 and 200 colonies per plate. Each sample was observed in triplicate (three plates were used to incubate and then the colonies counted) to ensure accuracy and reliability of the results. Finally, the number of formed colonies was registered.

### 2.8 Minimal inhibitory concentration

MIC was done for both cultures. A concentration scan is carried out, considering 8, 16, 100, 300, 1,000, and 2,750 μg/mL of PFL-OO and PFL-DMSO [maximal volume (0.5% v/v)], and OO with no drug were added to 1 mL of bacteria culture at the beginning of the exponential growth phase, approximately 1 × 10^8^ CFU/mL of cell density (Optical density of 0.02 A.U. at 600 nm). Then, in a broth plate with 96 wells, it was added 100 μL of bacterial culture with different concentrations of drugs and then incubated at 37°C for 12 h with gentle shaking. MIC values were obtained by measuring optical density at 600 nm to determine changes in turbidity.

### 2.9 Atomic force microscopy

Three samples of bacteria culture were cultivated independently with propofol at 2.75 mg/mL (final concentration). DMSO at 0.5% v/v was used as a carrier. Here, it is important to mention that, to correctly perform the AFM experiments, the carrier was changed because the oil nanodroplets stuck on the cantilver making the measurements difficult. This inconvenient did not show up when using DMSO. Since it was proved that the obtained effects are produced by the drug, nor by the carrier (see below), DMSO was a good choice. Bacteria strains were grown for 2 h at 37°C with 130 r.p.m., and then, 10 mL of cells were centrifuged at 4,000 r.p.m. for 10 min. The pellet was re-suspended in 10 mL of PBS buffer and centrifuged at 4,000 r.p.m. for 10 min, for three times. Finally, another three washes with deionized water were performed. 10 μL of each solution was poured onto a glass cover slip previously treated with 0.1% w/v poly-l-lysine. The drops were allowed to dry overnight to be measured the next day.

Measurements were made with an atomic force microscope (Innova Bruker, Santa Barbara, CA). The cantilever MSNL-10 was used. The tip spring constant was 0.03 N/m which has a resonant frequency around 15 kHz and radius of 2 nm. The images were taken in contact mode with a resolution and scan rate of 512 × 512 pixels and 3 Hz, respectively. The set points were: 4 V (for *E. coli*) and 2 V (for *S. aureus*), with proportional-integral-derivative (PID) parameters: 3,1,1. All measurements were performed at room temperature. Image processing was done using the Gwyddion 2.62 software.

### 2.10 Fluorescence microscopy

Three samples of bacteria culture were cultivated independently with propofol at 2.75 mg/mL (final concentration). DMSO at 0.5% v/v was also used as a carrier to avoid any interference with the measurements. Bacterial strains were grown for 2 h at 37°C and 130 r.p.m.; then, 10 mL of cells were centrifuged at 4,000 r.p.m. for 10 min. The pellet was re-suspended in 10 mL of PBS buffer and centrifuged at 4,000 r.p.m. for 10 min. This maneuver was done three times. Finally, another three washes with deionized water were performed. 200 μL of each solution was mixed with 10 μL of TR-DHPE (100 μg/mL); then, all solutions were incubated for 1.5 h in darkness at room temperature. An inverted fluorescence microscope (Axio Observed.Z1, Zeiss, Germany) was used to observe the interaction between the bacterial cell membrane and TR-DHPE dye. The filter used for TR-DHPE dye was L50 Cy5. Pixel sizes for the image are 4.54 × 4.54 μm, mode resolution 2,752 × 2,208, and exposure time of 600 ms. The images were processed using ZEN 2 Pro software.

### 2.11 Statistics

Three independent experiments with three replicates each were carried out. The replicates were averaged, and the resulting averages were subjected to the Shapiro–Wilk test to assess the normality of the distributions. The Shapiro–Wilk test revealed that the distributions were not normal. Therefore, we used the Wilcoxon rank test to determine significant differences between controls and experiments. The asterisk indicates a significant difference with a p-value ≤ 0.1.

## 3 Results and discussion

Before starting the experiments with the mentioned drugs, the stability of the nanoemulsions is verified by obtaining their size distributions and zeta potentials using dynamic light scattering (see details in Section 2.6). The suspensions were diluted by 10x before the measurements, to protect the electrodes of the zeta potential cells.

**Figure 1:**
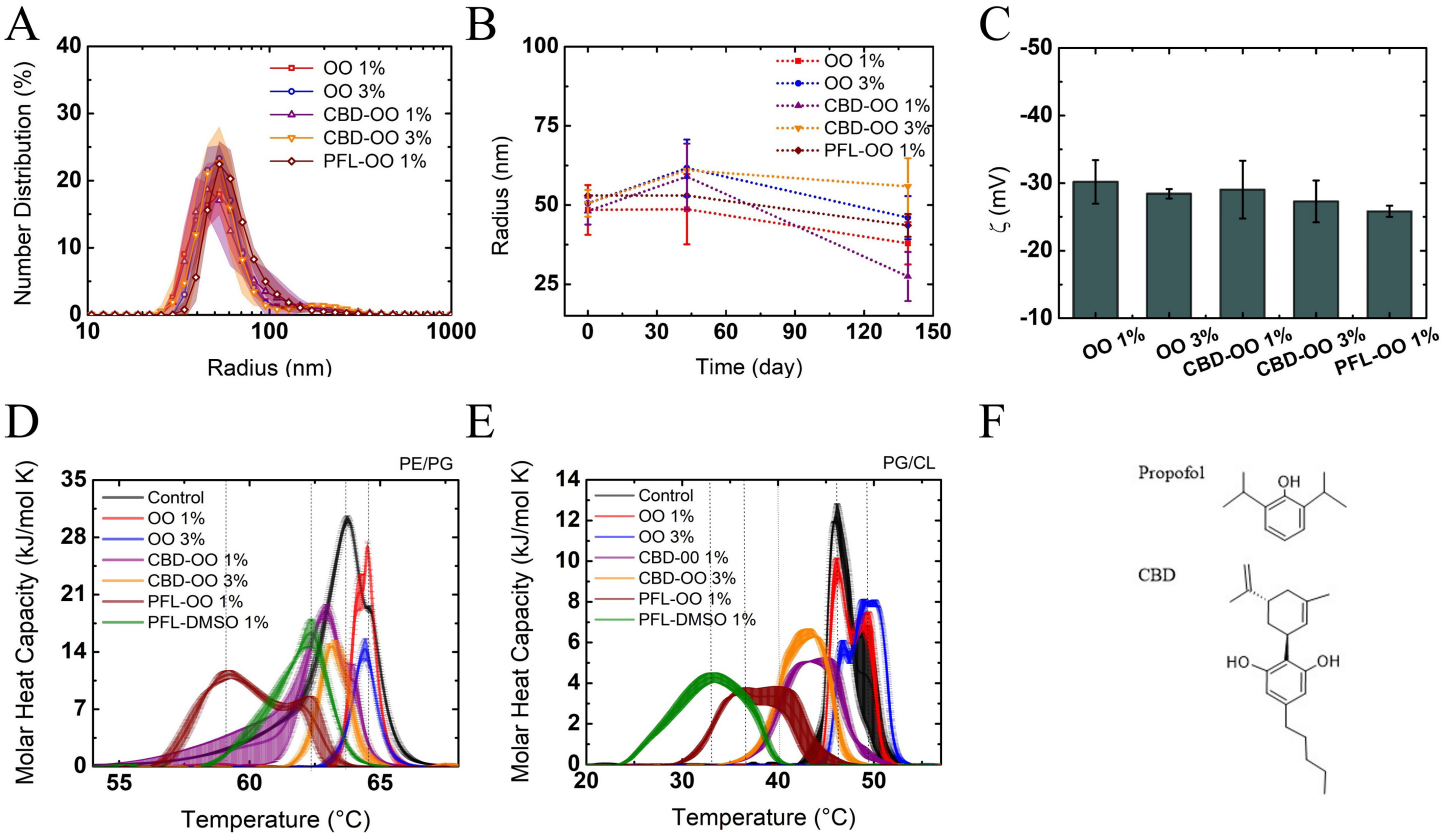
Characterization of the nanoemulsions through **(A)** size distribution, **(B)** kinetic stability, and **(C)** Zeta potential of the particles. The particle sizes and electric charge are very stable. Effects of nanoemulsions on liposomes using DSC: calorimetric profiles of *E. coli* **(D)** and *S. aureus* **(E)**-like-membranes. In both cases, PFL-OO 1% v/v has the greatest effect in the modification of cooperativity and transition temperature T_*m*_ with respect to the controls (black lines of profiles of pure liposomes) (see Table 1). Chemical structures of the evaluated drugs: propofol and CBD **(F)**.

All the size distributions of the nanodroplets were predominantly centered at approximately 50 nm (Figure 1A) and maintained their sizes for at least 15 days, which was enough time to perform the experiments (see Figure 1B). The negative zeta potential is the reason why coalescence is negligible (the droplets repel each other by an electrostatic force) (see Figure 1C). To explain the origin of the negative charge of the droplets, let us remember that olive oil is a composition of fatty acids such as linoleic, stearic, palmitic, and linolenic acids (Boskou et al., 2006). Since the pK of the carboxyl groups contained in their structure is very low (approximately 3.5), and the continuum phase of the nanoemulsion is deionized water (pH approximately 6), deprotonation occurs, being the cause of the negative z potential (Yang et al., 2013).

By evaluating the effect of the nanoemulsions on the model membrane of PE/PG as a *E. coli*-like-membrane and PG/CL as a *S. aureus*-like-membrane, it was noted that, compared to the calorimetric profiles of the control, the olive oil nanocarriers either produced a small shift to higher temperatures (due to the inclusion of the fatty acids in the membrane that increases molecular packing and therefore stiffness) (Figure 1D, or did not so, Figure 1E, see Table 1). Therefore, it can be presumed that they are harmless to the profiles and good choices for preparing the nanoemulsions. Indeed, the effects of PFL-OO and CBD-OO (1% v/v), and CBD-OO (3% v/v) are clearly opposite: The profiles stretch, deform, and shift at lower temperatures, indicating strong fluidization and loss of membrane cooperativity (Figures 1D, E). The shoulders in the profiles are associated with a phase separation due to the presence of different domains, regions where the drugs have not been distributed homogeneously (Korkmaz and Severcan, 2005). Although both drugs strongly affect the thermodynamic properties of the vesicles, and therefore compromised the integrity of the membranes, it is remarkably that PFL-OO 1% v/v has a stronger effect than CBD-OO 3% v/v. In fact, PFL-OO 1% v/v moved the T_*m*_ 5°C more than CBD-OO at 3% v/v (see Table 1). The widths of the profiles shown in Figures 1D, E were obtained by the standard deviations of three repetitions.

**Table 1:** Transition temperatures (temperatures of main peaks) and enthalpies (areas under the curves), with standard deviations, obtained from DSC profiles.

	**Enthalpy (kJ/mol)**	**STD**	**Tm (°C)**	**STD**
***E. coli*** **membrane-like lipids**				
Control	79.19	3.51	63.77	0.45
OO 1%	27.20	3.78	64.53	0.65
OO 3%	16.92	1.19	64.40	1.14
CBD-OO 1%	43.22	7.25	62.97	0.89
CBD-OO 3%	21.34	0.72	63.12	0.17
PFL-OO 1%	46.73	2.64	59.12	0.51
PFL-DMSO 1%	40.54	0.74	62.42	1.64
***S. aureus*** **membrane-like lipids**				
Control	42.79	9.26	46	0.42
OO 1%	36.82	1.63	46.31	0.54
OO 3%	39.38	0.28	49.28	0.09
CBD-OO 1%	39.11	0.11	43.93	0.08
CBD-OO 3%	39.92	0.12	43.35	0.23
PFL-OO 1%	33.79	5.94	36.55	0.18
PFL-DMSO 1%	40.77	3.01	33.30	0.24

**Figure 2:**
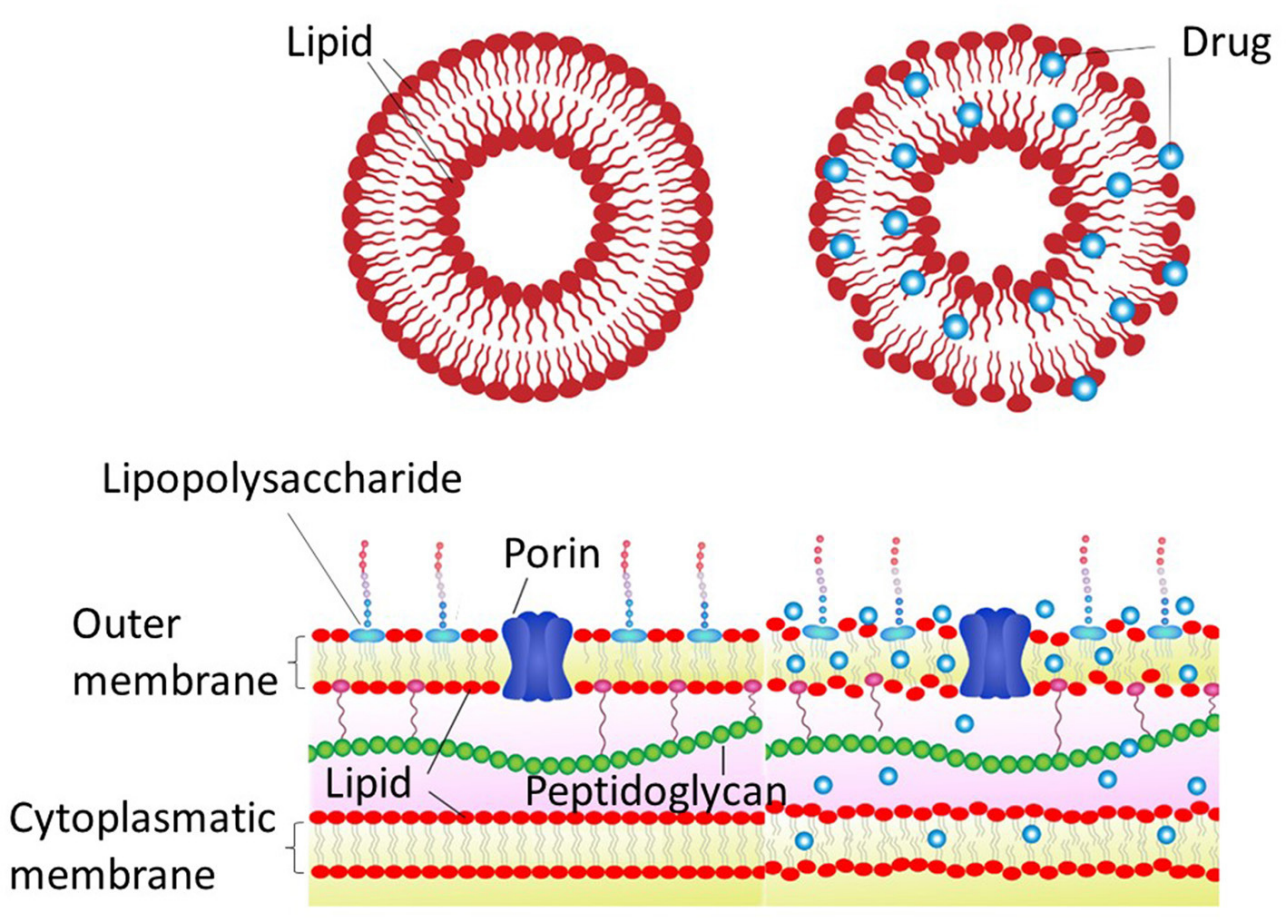
Schematic representation of the hydrophobic drug effects on the liposome model and bacterial membrane. The **top** panels depict a lipid vesicle before and after the drug insertion, and the **lower** panel schematizes a Gram-negative bacteria under such conditions.

To illustrate the calorimetric changes described above using a simplistic scheme, the cartoon depicted in Figure 2 may be useful. Indeed, the observed changes in the cohesion enthalpies produced by the drugs give rise to a general disorder in the membranes.

It is important to remark that the effects generated by CBD and PFL (see their molecular structures in Figure 1F) on both PE/PG and PG/CL are driven by enthalpic and entropic forces. This indicates that the intrusion of external molecules into the bilayer affects the cohesive energy. Since the 1% v/v and 3% v/v nanoemulsions in the vesicle suspensions corresponded to 2.75 mg/mL (1% v/v) or 8.25 mg/mL (3% v/v), respectively, propofol has a higher number of molecules owing to its lower molecular weight, which could explain its stronger effect on the membrane integrity. However, it is important to note that these concentrations translate into 15.42 mM for PFL-OO (1% v/v), and 8.74 and 26.22 mM for CBD-OO (1% v/v and 3% v/v), respectively. As a result, the amount of molecules is not the unique reason for its greater effect. In addition, since CBD is much more hydrophobic than PFL, hydrophobicity is not the reason for the effect either.

The disrupting effect of PFL has been widely studied before (Pérez-Isidoro et al., 2014; Paiva et al., 2012). Dynamic molecular simulations revealed that PFL exhibits a preference for localizing near the hydrocarbons tails of the phospholipids (Hansen et al., 2013). There, the hydroxyl group of the propofol interacts with their ester oxygens, generating perturbations that cause the lipids to go through the gel-liquid phase with less energy. Similarly, the CBD molecule contains two hydroxyl groups that can interact with the ester oxygen, and the rest of the molecule is oriented toward the hydrophobic region. Furthermore, it is important to note that the drugs were carried by nanodroplets. Consequently, adding hydrophobic molecules to the dispersed phase (olive oil) prevents them from dissolving in the continuous phase (water). Yet, PFL could diffuse better than CBD to reach the lipid bilayer. In addition, it has been shown that, despite being more soluble in octanol and therefore more likely to diffuse into lipid membranes, molecules with logP greater than 5 have reduced penetration in cells (Lipinski et al., 2012).

If a simple membrane model like the one discussed above undergoes a significant change in its structural condition when interacting with PFL and CBD, which completely modify the transition temperature and enthalpy of cohesion, as shown in Figures 1D, E, a biological membrane may also suffer similar modifications in its membrane integrity, compromising the organism viability (see Figure 2).

**Figure 3:**
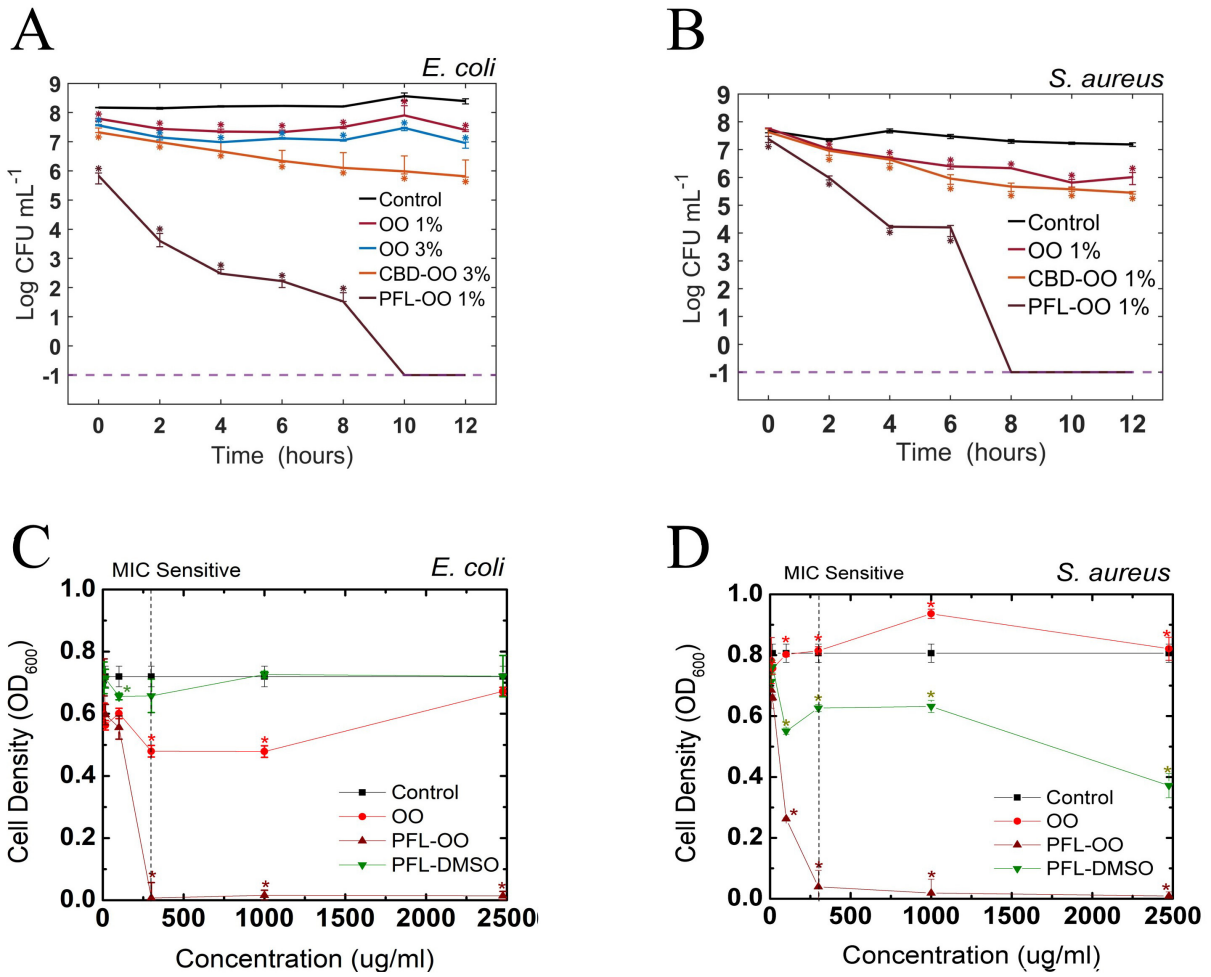
Effect of various agents on *E. coli* and *S. aureus* bacteria with a final concentration of 24–10^8^ CFU/mL (Optical density of 0.04 A.U. at 600 nm). **(A)** For *E. coli*, we show that the vehicles (OO 1% v/v and OO 3% v/v) have the same small effect. Propofol-OO has a full bactericide effect, while CBD-OO needs to be augmented to 3% v/v to give rise to a 99% in the decrease of viability (2log reduction). In the vertical scale, –1 indicates that no bacteria survived after 10 h. **(B)** For *S. aureus*, the antimicrobial effectiveness of propofol is even better since –1 is obtained at 8 h. The minimum inhibitory concentration (MIC) of nanoemulsions (OO and PFL-OO) and PLF-DMSO was found in both bacteria after 12 h of cell culture growth **(C, D)**. Three independent experiments were performed in our experiments. The median and the interquartile values in the error bars are shown. Asterisks mean the difference statistics tested with the Wilcoxon rank.

To test this hypothesis, the effect of the nanoemulsion on both bacteria was evaluated by assessing the growth behavior of colonies in agar plates. This assay consisted in proving first the harmless effect of the vehicle and then performing independent test of the bactericide effects of the drugs (see Figures 3A, B). To see whether the incubation time was an important factor, the CFU was measured at different periods of time. It is clear that propofol, prepared at 1% v/v concentration, has a fully annihilation effect in *E. coli* after 10 h and in *S. aureus* after 8 h. On the other hand, CBD at 1% v/v produced only a mild inhibition effect on the growth of bacteria compared to the control, but at 3% v/v reduced a bit more than two decades in a log scale, which means a drop in viability better than 99%.

Our findings contrast with previous studies on propofol emulsions, where it has been reported that they promote bacterial growth when dissolved in soybean oil. Hence, it seems that nanoemulsions are susceptible to contamination (Baker et al., 2005). For such a reason, efforts to reformulate the colloidal suspensions have been undertaken, for example, the use of other oily vehicles and/or the addition of ethylenediaminetetraacetic acid (EDTA) (Sakuragi et al., 1999; Wang et al., 2007; Shevalkar et al., 2019). In contrast, our results showed that, carried by olive oil nanodroplets, PFL at 1% v/v strongly inhibits the growth of *E. coli* and *S. aureus* bacteria.

Some action mechanisms have been studied for the bactericidal action of hydrophobic molecules (Blaskovich et al., 2021). It is proposed that one of the potential unexplored targets could be the composition of the outer lipid bilayer because the membrane is the first component found between the extracellular medium and the cells. Indeed, to understand our findings beyond the usual conception behind antibiotic strategies, which are mostly based on attacking specific biochemical targets, or in creating membrane pores that shortcut the membrane potential, it is important to discuss a recent paper by some of us (Salinas-Almaguer et al., 2022). It was studied the effect produced by pentanol as a membrane softening by bilayer disruption. Similar to the damage produced by fluidization (Royce et al., 2013), or lethal permeability upsurge (Vaara, 1992), it is shown that, after a long induction phase in which the microorganisms are striving against a bacteriostatic action, the cells cannot proliferate due to lipid packing distortion which results in further delocalization of membrane proteins impairing multiple essential processes including respiration (Strahl and Hamoen, 2010), nucleotide synthesis (Wenzel et al., 2014), FtsZ assembly (Silber et al., 2020; Mileykovskaya et al., 1998), and nucleoid segregation (Mileykovskaya et al., 1998; Chai et al., 2014), with the collateral effect to harm the cytokinetic apparatus so as to prevent and/or delay the membrane constriction need for normal cell division and offspring generation. In summary, a membrane disruptor such as pentanol, or propofol, compromises the crucial role of membrane rigidity as the key regulator of bacterial proliferation. Other studies of essential oil nanoemulsions have found toxic effects on bacteria (Moghimi et al., 2016; Donśı et al., 2012; Terjung et al., 2012), although the mechanisms involved are attributed to perturbation and cellular leakage caused by the hydrophobic components of the nanodroplets (Seow et al., 2014).

As above mentioned, since PFL is much less hydrophobic than CBD, it can be more easily solubilized in the medium. After the exogenous molecules are internalized in the membrane, they alter its correct functioning. The greater their ability to diffuse in the membrane, the greater the cellular damage inflicted.

Since it is crucial to investigate whether a lower dose could give similar results in terms of bacterial inhibition, experiments to find the minimal inhibitory concentration (MIC) were performed. First, the results show that the most effective carrier was PFL-OO, compared to PFL-DMSO (see Figures 3C, D). Second, the MIC sensitivity in both bacteria was 300 μg after 12 h of incubation, much lower than the concentration used above in the CFU assay (2.75 mg/mL). Let us remark though that MIC assays are performed in liquid media Luria-Bertani (LB) Broth and the CFUs on LB agar.

**Figure 4:**
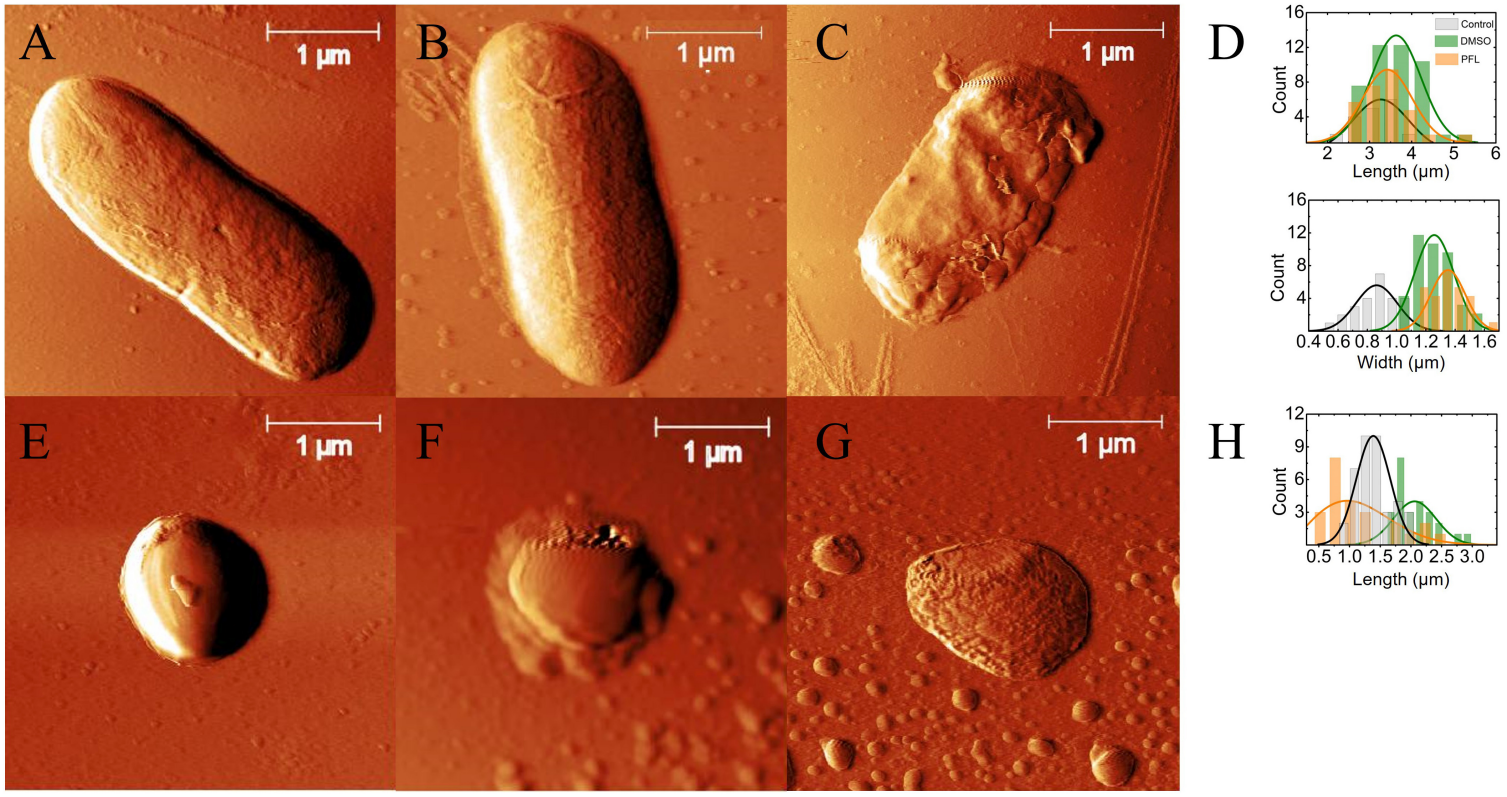
*E. coli* and *S. aureus* top images obtained by AFM. **(A, E)** with no treatment (controls), **(B, F)** with DMSO at 0.5%, and **(C, G)** with DMSO/PFL; *E. coli* and *S. aureus*, respectively. The images illustrate the dramatic damages caused in the cell membranes by PFL. Lengths and widths of the cells: for *E. coli* **(D)** and for *S. aureus* **(H)**. In the case of *E. coli*, the histograms were obtained from 21 (no treatment), 43 (DMSO), and 27 (PFL) cells. For *S. aureus*, the histograms were calculated from 39 (no treatment), 25 (DMSO), and 25 (PFL) cells.

As a crucial probe to confirm that the morphology of bacteria after the treatment is similar to the one depicted in Figure 2, AFM images were captured as shown in Figure 4. Let us remark that we focused only on the effect produced by propofol, which was the drug that produced the greatest effects on the viability of the organisms.

**Figure 5:**
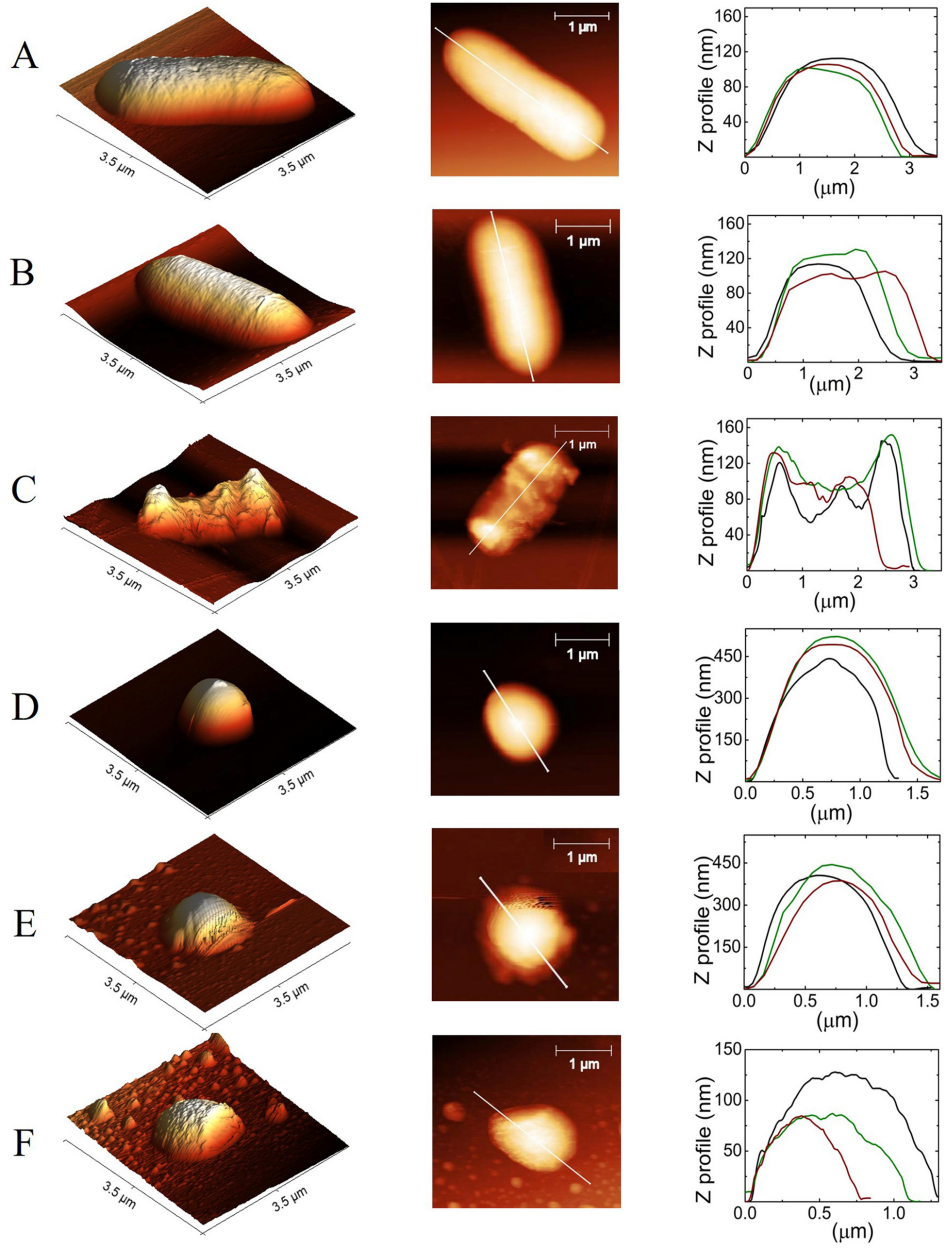
Contact-mode height AFM images of *E. coli* and *S. aureus*. **(A, D)** Show control cells, **(B, E)** after treatment with DMSO at 0.5%, and **(C, F)** after DMSO/PFL treatment, *E. coli*, and *S. aureus*, respectively. The first column corresponds to 3D topographic images, the second corresponds to 2D, and the third column corresponds to the height and size measurement of the selected cross-sectional line of the image. Black, green, and red lines are repetitions for three different bacteria. Despite the dispersion, it is clear the effect of PFL.

In Figures 4A, E, it is observed the typical rod-shaped and spherical structures of *E. coli* and *S. aureus*, whose average sizes are shown in Figures 4D, H, which is in agreement with previous reports (Meincken et al., 2005; Yao et al., 2012; Harris and Theriot, 2016). Likewise, the cells with DMSO did not exhibit great deformations on their membranes, indicating that DMSO (0.5% v/v) was not harmful (see Figures 5B, F). It is important to remark that the growth of the colonies in the experiments reported in Figure 3 was not assessed, not only because so little concentration of DMSO is innocuous (Lim et al., 2012; Summer et al., 2022), but also because the evolution of the optical density for both bacteria, with and without DMSO, was the same.

The cells with PFL were notoriously different, as observed in Figures 4C, G. It is obvious that these changes in the membrane compromise the viability of the cells, explaining the results shown in Figure 3. Therefore, the mechanistic reason for bacterial death is simply that this hydrophobic molecule completely affects the entire membrane, inflicting drastic damages in the regulation of bacterial proliferation.

It is enthralling that *S. aureus* bacteria treated with DMSO/PFL not only lose the spherical morphology but also give rise to small vesicles not observed in the control sample, probably due to homeostatic and stressful conditions. Indeed, to combat stressors and survive, pathogens have established various defensive mechanisms, and one of them is the production of membrane vesicles (Mozaheb and Mingeot-Leclercq, 2020).

Figure 5 shows topographic images corresponding to Figure 4. It is clear the expected shape of both bacteria in A, B, D, and E (Chang and Liu, 2018; Salinas-Almaguer et al., 2015; Whitehead et al., 2006). Next, it is shown that PFL produces height irregularities in *E. coli* (C) and *S. aureus* (F). The side profile along the length of the bacterium, as shown in the second column, is consistent with such 3D images. The height (Z) profiles from the organisms are seen in the last column, which indicates a non-random behavior. Note the dramatic change in the topography of the cell membrane with PFL treatment along the cross section, which modifies shape and height. It is worth mentioning that the very low concentration found in our MIC experiments could produce damage to the bacteria membranes in the same way as those shown in Figure 4, although not necessarily observable with the sensitivity of our AFM.

**Figure 6:**
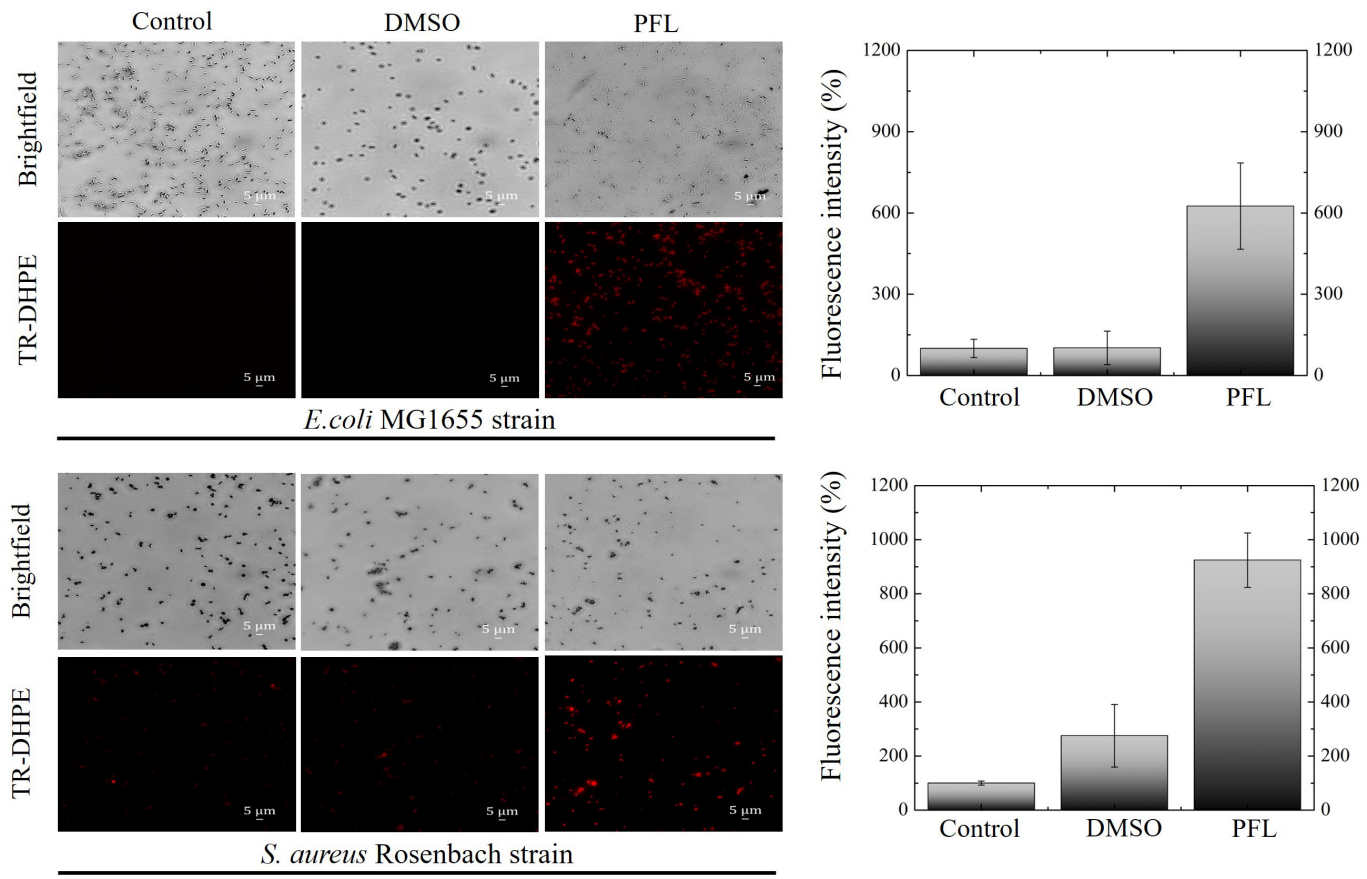
Fluorescence images of the effects of propofol on the bacterial membrane of *E. coli* and *S. aureus* cultures after 1.5 h treatment. The red fluorescence indicates that the phospholipid (TR-DHPE) penetrates the cell membranes. Clearly, in both cases, inward diffusion is promoted by the membrane disruption caused by propofol. Three groups are shown: control, drug carrier (DMSO), and drug (PFL). A 40X objective was used.

As a final test to evaluate bacterial membrane damage caused by PFL, it was performed fluorescence microscopy measurements, using a phosphoethanolamine dye (TR-DHPE), which is employed as an indicator for liquid disordered membranes (Krivic et al., 2022; Skaug et al., 2011; Baumgart et al., 2007; Veatch and Keller, 2005). We evaluated the effect in both culture strains (see Figure 6). Brightfield and fluorescence images are depicted. It is clear that the dye diffuses in both bacteria only when they are treated with PFL, although in the case of *S. aureus*, the dye penetration is stronger. Even in the absence of PFL (empty DMSO carrier), the dye diffuses a little. This observation is consistent with the fact that *S. aureus* does not have an additional barrier. Furthermore, this also explains the greater inhibition produced by PFL for the Gram-positive bacteria (see Figures 3A, B).

It is important to note before closing that the research we report here is not the first to explore the promising idea that hydrophobic drugs can be used as a treatment against bacteria. In fact, growth inhibition has been reported to occur for *S. epidermidis, S. pyogenes*, and *S. pneumoniae* with 2.5 mg/mL of the local anesthetic bupivacaine (Rosenberg and Renkonen, 1985). Furthermore, it has been shown that nanoemulsions can significantly improve the efficacy of the hydrophobic drugs delivery, as demonstrated with the carbapenem-resistant *K. pneumoniae* (Tayeb et al., 2022). However, to the best of our knowledge, our study is the first to explore pathogen membrane destabilization upon insertion of hydrophobic drugs. We found that PFL has a remarkable effect in this regard.

To put our findings in perspective, we claim that PFL, and to a lesser extent CBD, could have enormous potential in skin infections where antimicrobial resistance is already a medical problem. In fact, despite recent advances in wound treatment, very few topical therapies have proven effective in promoting wound healing, especially because when recalcitrant bacteria invade wounds, they create a cytotoxic environment that often promotes very serious injuries (Kawano et al., 2020). Furthermore, it is worth mentioning that the method proposed here could be combined with other strategies to improve effectiveness. For example, since essential oils have been found to enhance the antimicrobial activity of drugs and also inhibit the transmission of resistance to other populations (Bueno, 2016), our nanoemulsions could be used in combination with antibiotics or antimicrobial peptides (AMP). Clearly, they could act as adjuvants to increase the uptake of the antibiotic through the bacterial membrane in topical applications. The absorption of PFL through the skin, if it occurs, would not be a problem, since the concentration used here (2.75 mg/mL) is less than the injectable dose normally administered during anesthesia (Schüttler and Ihmsen, 2000). In fact, for a person who weighs approximately 70 kg, the propofol infusion rate is between 2 and 4 mg/mL per minute, for not less than 60 min (time for rapid surgery). This is equivalent to at least 120 mg. Furthermore, recent investigations on the anesthetic effects of propofol through skin absorption found that this drug may have a potential use in clinical practice, especially in pediatric applications (Zhang et al., 2021), where the concentrations used are approximately 10 mg/mL. Concomitantly, this transdermal way of administering propofol gels implies safety for cutaneous/topical applications.

Two limitations of this study are the unexpected poor outcome of CBD and the absence of evidence on the possible medical application of our findings. As we expected the more hydrophobic CBD to give better results but it did not, we would like to provide a positive electrical charge to the nanodroplets and make the release of the drug into the bacterial membranes, which have a negative charge, more efficient. In addition, we would like to test PFL in model animal infections. Such a strategy could provide us with important information about medical applications in humans, to try to contribute to the imminent health emergency that is approaching.

## 4 Conclusion

In the present study, hydrophobic nanoemulsions based on olive oil as potential inhibitors of bacterial growth were investigated. We found that propofol has a greater effect than CBD to annihilate bacteria, owing to its property to alter their membrane integrity. Beyond their ability of survival, the pathogens cannot cope with the stress produced by hydrophobic molecules that diffuse in their membranes compromising the cells to proliferate. This study could help in the search for new antimicrobial drugs whose action is neither selective nor on a specific target but directed non-specifically at cell membranes.

## Data Availability

The original contributions presented in the study are included in the article/supplementary material, further inquiries can be directed to the corresponding author.
